# Bayesian approach to investigate a two-state mixed model of COPD exacerbations

**DOI:** 10.1007/s10928-019-09643-6

**Published:** 2019-06-13

**Authors:** Anna Largajolli, Misba Beerahee, Shuying Yang

**Affiliations:** 1 GlaxoSmithKline, Research and Development, Uxbridge, UK; 2 Present Address: Certara Strategic Consulting, Via G.B. Pirelli 27, 20124 Milano, Italy; 3 Clinical Pharmacology Modelling and Simulation, Quantitative Sciences, GlaxoSmithKline, Stockley Park West, 1-3 Ironbridge Road, Uxbridge, Middlesex, UB11 1BT UK

**Keywords:** Bayesian, Two-state model, Negative Binomial, Exacerbations, COPD

## Abstract

Chronic obstructive pulmonary disease (COPD) is a chronic obstructive disease of the airways. An exacerbation of COPD is defined as shortness of breath, cough, and sputum production. New therapies for COPD exacerbations are examined in clinical trials frequently based on the number of exacerbations that implies long-term study due to the high variability in occurrence and duration of the events. In this work, we expanded the two-state model developed by Cook et al. where the patient transits from an asymptomatic (state 1) to a symptomatic state (state 2) and vice versa, through investigating different semi-Markov models in a Bayesian context using data from actual clinical trials. Of the four models tested, the log-logistic model was shown to adequately characterize the duration and number of COPD exacerbations. The patient disease stage was found a significant covariate with an effect of accelerating the transition from asymptomatic to symptomatic state. In addition, the best dropout model (log-logistic) was incorporated in the final two-state model to describe the dropout mechanism. Simulation based diagnostics such as posterior predictive check (PPC) and visual predictive check (VPC) were used to assess the behaviour of the model. The final model was applied in three clinical trial data to investigate its ability to detect the drug effect: the drug effect was captured in all three datasets and in both directions (from state 1 to state 2 and vice versa). A practical design investigation was also carried out and showed the limits of reducing the number of subjects and study length on the drug effect identification. Finally, clinical trial simulation confirmed that the model can potentially be used to predict medium term (6–12 months) clinical trial outcome using the first 3 months data, but at the expense of showing a non-significant drug effect.

**Electronic supplementary material:**

The online version of this article (10.1007/s10928-019-09643-6) contains supplementary material, which is available to authorized users.

## Introduction

Chronic obstructive pulmonary disease (COPD) is defined in the Global Initiative for chronic obstructive lung disease (GOLD) report as a common preventable and treatable disease, characterized by persistent airflow limitation that is usually progressive and associated with an enhanced chronic inflammatory response in the airways and the lung to noxious particles or gases [1]. COPD is a leading cause of morbidity and mortality worldwide and results in an economic and social burden that is both substantial and increasing. Exacerbations and co-morbidities contribute to the overall severity in individual patients.

An exacerbation in COPD is defined as an acute event characterized by a worsening of the patient’s respiratory symptoms (e.g. dyspnoea) and non-respiratory symptoms (e.g. fatigue) that is beyond normal day-to-day variations and leads to a change in medication or even hospitalization [2].

The latest GOLD guidelines point out, the yearly exacerbation rate is an important risk factor to consider as it affects the patient’s health status and its lung function over time [1]. Reducing the number of exacerbations would have a beneficial impact on the patient life and on the disease status.

Exacerbations are historically analysed in terms of frequency through the negative binomial approach that assumes that for each individual the number of exacerbations can be described by a Poisson process with a different rate for each subject [3].Due to the high variability in occurrence and duration of the event, longer term clinical study and large sample size are needed to observe enough exacerbations in the trials to detect meaningful treatment effect. Moreover, a frequency analysis ignores the variability in the number and the duration of each exacerbation among individuals. Figure S1 shows some example patterns of few individuals’ exacerbations represented as duration in asymptomatic state (state 1) and symptomatic (exacerbation, state 2): it is evident that some participants experience shorter duration exacerbations while others may have a few but long duration exacerbations.

Like many other chronic diseases such as chronic bronchitis, migraine and psychiatry, COPD patients’ experience of exacerbations can be described as subjects making multiple transitions between asymptomatic and symptomatic state.

Ng and Cook [4] and Cook et al. [5, 6] characterized such exacerbations data in terms of a two-state stochastic process where the patient alternates between an asymptomatic state or state 1 (i.e. no exacerbation is present) and a symptomatic state or state 2 (i.e. where exacerbation is present) under the first order Markov assumption that says that the next state depends only on the present state and not on the history of the process. In this way, not only the information of exacerbation is considered but also the time that the patient spends in each state: therefore, a more complete use of the information present in the dataset is achieved.

In this work, we investigated this two-state approach in Bayesian context, and aimed to extend Cook’s approach by exploring different Markov and semi-Markov distribution assumptions (i.e. respectively exponential model and Gompertz, log-logistic model) and by comparing them with the simpler semi-Markov assumption investigated by Cook et al. (i.e. Weibull model). Note that using Bayesian approach allows no assumptions on model parameters and the semi-Markov feature implies that the memory of the system changes with time, conversely from the Markov feature that implies constant time dependency. Several clinical trial data with different mechanisms of treatments and study duration were used to evaluate performance of the models in identifying drug effect.

## Materials and methods

### Data

#### Data 1

A 52-week Phase III placebo controlled trial that evaluates the efficacy and safety of the inhaled Salmeterol/Fluticasone Propionate combination product (50/500 mg strength) twice daily with Salmeterol 50 mg twice daily alone and Fluticasone Propionate 500 mg twice daily alone (GSK study ID: SFCB3024). Subjects who were diagnosed with mild, moderate, severe COPD according to the ATS staging system [7] and had at least 10 pack-years of smoking history and at least one acute COPD exacerbation per year in the past 3 years were included in the study. For this work, data from the Salmeterol treatment alone or placebo were used.

Total of 619 subjects administered with Salmeterol or placebo were included in this analysis.

#### Data 2

Two replicate 52-week Phase III studies that evaluate the effects of once daily inhaled treatment with three dosage strengths of fluticasone furoate “FF”/vilanterol “VI” (FF/VI) (50/25, 100/25 and 200/25 mcg) versus one dosage strength of VI (25 mcg) in subjects with COPD (HZC102970/NCT01017952, HZC102871/NCT01009463).

From these studies, we included 3238 subjects that were diagnosed with moderate, severe and very severe COPD according to GOLD staging system [1] in this analysis.

#### Data 3

A phase 2b placebo-controlled study that evaluates the efficacy and safety of the oral dosing Losmapimod, an experimental anti-inflammatory drug, administered twice daily, that is compared (2.5, 7.5 and 15 mg) with placebo for 24 weeks in adult subjects with COPD (MKI113006/NCT01218126).

Total of 602 subjects that were classified according to GOLD standard mainly Stage II or Stage III were included in the analysis. In addition, the similar analysis was also conducted in a sub-group with low eosinophils (EOS) as findings in [8] indicate eosinophil-related heterogeneity within COPD and suggest that losmapimod could be a potential therapy to reduce exacerbations in COPD patients with eosinophil levels ≤ 2%.

Note that each study was approved by a national, regional, or investigational centre ethics committee or institutional review board, in accordance with local requirements. Each patient gave written informed consent before enrolment.

Table 1 presents the description of the three datasets available for this work and the key covariates of interest in each dataset. Further details on the conduct and primary results of the studies are available via ClinicalTrials.gov.

**Table 1:** Demographic information of the three datasets used

	Data 1			Data 2			Data 3		
	Treatment	Placebo	Sum	Treatment	Placebo	Sum	Treatment	Placebo	Sum
Smoke status									
Unknown	4	3	7						
Current-smoker	156	142	298	1064	363	1427	192	72	264
Ex-smoker	155	159	314	1359	452	1811	257	81	338
Disease stage									
Missing				22	8	30			
Mild	143	140	283	1	0	1	3	1	4
Moderate	110	114	224	951	305	1256	176	52	228
Severe	62	50	112	1103	383	1486	200	77	277
Very severe				346	119	465	70	23	93
Total	315	304	619	2423	815	3238	449	153	602
Pack year smoking (current smokers)									
Mean	45.9	41.8							
SD	18.4	17.3							
Min	12	11.8							
Max	99.8	90							
Season									
Autumn–winter	142	133	275						
Spring–summer Mar–Aug	173	171	344						
Total	315	304	619	2423	815	3238	449	153	602

For Data 1, the disease stage was classified according to ATS 1995 guideline (%predicted FEV1 ≥ 50 = mild; 35–50 = moderate; < 35 = severe); for Data 2 and Data 3, the disease stage was classified according to GOLD guideline (%predicted FEV1 ≥ 80 = mild; 50–80 = moderate; 30–50 = severe; < 30 = very severe)

### Two-state semi Markov model

Ng and Cook [4] and Cook et al. [5, 6] were first to propose this approach in a bronchitis trial to describe the exacerbation process but generally this method can be extended to various chronic diseases in which subjects make multiple transitions between asymptomatic and symptomatic state (e.g. in infectious diseases, neurology and rheumatology as described in Cook et al. [6]). In this work, we explored different semi-Markov distribution assumptions to describe the transition (and the non-transitions as well in case of subjects that do not change state) of COPD exacerbations.

Specifically, the onset and the resolution of the exacerbations were modelled through a two-state mixed semi-Markov renewal process where the sojourn time in a state depended on the time since entry into that the state. The transition rate of the jth subject from state k (k = 1, 2) was defined in the following way:

1 $$\lambda_{kj} = h\left( {t,p_{kj} ,\beta_{kw} |X_{j} } \right)$$

where h(t, ***p***_kj_, $\beta_{\text{kw}} |X_{\text{j}}$) was the hazard function that was a function of time, a set of parameters ***p***_kj_ associated with the renewal process and β_kw_(w = 0,…,M) that were the coefficients associated with the covariates (X_j_) that can be used to explain some of the variabilities. Note to account for the variation in duration that individuals might stay in the study, the study length for each individual was included as a covariate in the model.

The following four frequently used distribution functions [composed of a hazard ($h\left( {t,p_{kj} } \right)$) and survival function ($S\left( {t,p_{kj} } \right)$)] were investigated:

1. The exponential model

2 $$f_{kj} \left( {t,p_{kj} } \right) = h\left( {t,p_{kj} } \right) \times S\left( {t,p_{kj} } \right) = \theta_{kj} \times e^{{ - \left( {\theta_{kj} \cdot t} \right)}}$$

where $\theta_{kj}$ (> 0) is the scale parameter;

2. The Weibull model

3 $$f_{kj} \left( {t,p_{kj} } \right) = h\left( {t,p_{kj} } \right) \times S\left( {t,p_{kj} } \right) = \theta_{kj} \alpha_{k} \left( t \right)^{{\alpha_{k} - 1}} \times e^{{ - \left( {\theta_{kj} \cdot t^{{\alpha_{k} }} } \right)}}$$

where θ_kj_ (> 0) is the scale parameter, α_k_(> 0) is the shape parameter for state k;

3. The Gompertz model

4 $$f_{kj} \left( {t,p_{kj} } \right) = h\left( {t,p_{kj} } \right) \times S\left( {t,p_{kj} } \right) = \theta_{kj} e^{{\alpha_{k} \cdot t}} \times e^{{\left( {\frac{{\theta_{kj} }}{{\alpha_{k} }} \cdot \left( {1 - e^{{\alpha_{k} \cdot t}} } \right)} \right)}}$$

where θ_kj_ (> 0) is the scale parameter, α_k_ (− ∞, + ∞) is the shape parameter (https://cran.r-project.org/web/packages/reliaR/reliaR.pdf);

4. The log-logistic model

5 $$f_{kj} \left( {t,p_{kj} } \right) = h\left( {t,p_{kj} } \right) \times S\left( {t,p_{kj} } \right) = \frac{{\alpha_{k} \left( {t/\theta_{kj} } \right)^{{\alpha_{k} }} }}{{t \cdot \left( {1 + \left( {\frac{t}{{\theta_{kj} }}} \right)^{{\alpha_{k} }} } \right)}} \times \frac{1}{{\left( {1 + \left( {\frac{t}{{\theta_{kj} }}} \right)^{{\alpha_{k} }} } \right)}}$$

where θ_kj_ (> 0) is the scale parameter, α_k_ (> 0) is the shape parameter (http://www.openbugs.net/Manuals/Reliability/Manuals/Distributions.html#Log-Logistic).

These models differ in the assumptions on their hazard or survival functions (or a transformation of these functions) with time. Note that the exponential model has a hazard that is constant with time, the Weibull model has the logarithm of the hazard that depends linearly on the logarithm of time, the Gompertz model has the logarithm of the hazard that depends linearly on time whereas the log-logistic model has the logit of its survival that depends linearly on the logarithm of time.

Note that potential covariates effects (β_kw_) and random effects η_kj_ can be introduced in the parameters of the above model distributions in the following way:

6 $$\theta_{kj} = \theta_{{0_{kj} }} \eta_{kj} e^{{\left( {\log \left( {T_{j} } \right)\beta_{k0} + \beta_{k1} X_{1} + \ldots + \beta_{kM} X_{M} } \right)}}$$

$$\alpha_{kj} = \alpha_{{0_{kj} }} \eta_{kj}$$

where Tj was the study length for subject j.

Both scale and shape parameters were initially included in the models as fixed effects, and then random effects were included on top of the fixed effects as independent normal distributed variables. For the best model, where possible, correlation of the random effects was tested by assuming random effects followed a multivariate normal distribution.

Note that the covariate analysis was run using only Data 1 as it was the available dataset richer in covariates (Table 1). The following available covariates were tested for statistical significance namely, baseline seasonality, disease stage (ATS), smoke and yearly packs of cigarettes. Note that the last covariate was considered continuous whereas the first three covariates were first transformed into binary values 0 versus 1 [i.e. spring–summer (April–September) vs autumn–winter (October–March); disease stage mild vs rest; ex-smoker vs rest] to test their significance and then into more complex values re-parameterization only if the simpler binary parameterization resulted in a significant effect.

### Dropout

The impact of dropouts was also explored. The dropout was formalised as a time to event model. The drop out model was introduced to the two-state model to describe the probability of dropout at a certain time. It was assumed that drop out was missing at random which implies the dropout independency from the exacerbations data. Note that the criterion to define a subject that drops out is not strictly related to the clinical trial study length as the subject last visit can oscillate up to 1 month before the end of the entire study where the subject can stop to be monitored and at the same time not be defined a dropout.

The choice of the potential dropout model was investigated separately from the two-state model (i.e. using only dropout data) through three different parametric models: the exponential model that assumed a constant probability of an event over time, the log-logistic and the Weibull models that instead introduced a time dependency. Also the effect of the following covariates on the best dropout model: the number of exacerbations, the disease stage and total duration in state 2 were tested. The covariates were introduced in the similar way as that described in Eq. 1. Once the dropout model was optimized, it was integrated in the final two-state model and the performance of the integrated models was further investigated.

### Two-state model evaluation

To evaluate the performance of the two-state models, different simulation based diagnostics were used such as posterior predictive check (PPC) implemented as in Yano et al. [9] and visual predictive check (VPC) implemented as in Holford et al. [10]. Specifically, simulations of 100 datasets were performed using the final model. For building the PPC plots, different statistics were calculated on the simulated data and compared to that obtained in the observed data. For the VPC plots, the distribution of the duration in each state from each simulation was compared to the distribution of the observed duration. Note that the choice of the number of bins in the VPC was done by applying a clustering technique (i.e. k-means cluster as implemented in the R software [11]) based on the observed durations in each state that enabled us to find the compromise between the number of bins and the information carried in each bin through number of clusters vs sum of residuals plot. The final VPC binning was then obtained using the same clustering technique with the chosen number of bins.

Note that before diving into the simulation-based model diagnostics, the standard criteria to evaluate the model convergence in a Bayesian framework were first inspected (e.g. parameter trace plots, parameter posterior distributions).

### Drug effect evaluation and design consideration

Once the best model structure was identified, the drug effect was added and evaluated in all three datasets by looking at both the drug effect estimates and their relative confidence intervals and the transition rates and probability.

A further design investigation was performed to show the impact of the number of subjects and study length on the drug effect identification. It is noted that the subjects were randomly selected from the original data in order to have a sample through sampling without replacement that is still representative of the COPD population.

Finally, a validation analysis was done to evaluate the ability of the model to predict clinical trial data and the drug effect using relatively less amount of information (i.e. only 3 month data).

To evaluate the drug effect and different design settings the transition probability and the transition rate ratio [placebo vs active transition rates (see Eq. 1)] were considered.

### Bayesian analysis

A Bayesian method with vague priors on the model parameters [θ or α ~ normal (0,1000), β ~ normal(0,1000) and variance of η (σ^2^ = 1/τ) τ ~ gamma(0.001, 0.001)] was adopted in this work. Markov Chain Monte Carlo (MCMC) technique as implemented in OPENBUGS 3.2.3 rev 1012 [12] was used for all the analyses, to estimate the unknown parameters and to obtain their posterior distributions. In addition, instead of using the standard distributions in the library, the general dloglike function (http://www.openbugs.net/Manuals/Tricks.html#GenericDistribution) in OPENBUGS was applied, as it enabled us to implement explicitly the transition rate distribution function together with the censoring information when needed. When simulations were performed, OPENBUGS was called from the R [11] (version 3.2.3) package BRugs. In addition, the deviance information criterion (DIC), a Bayesian measure of model fit that penalizes model complexity (the smaller the value the better the fit of the model), was used to assist the model selections.

## Results

**Fig. 1:**
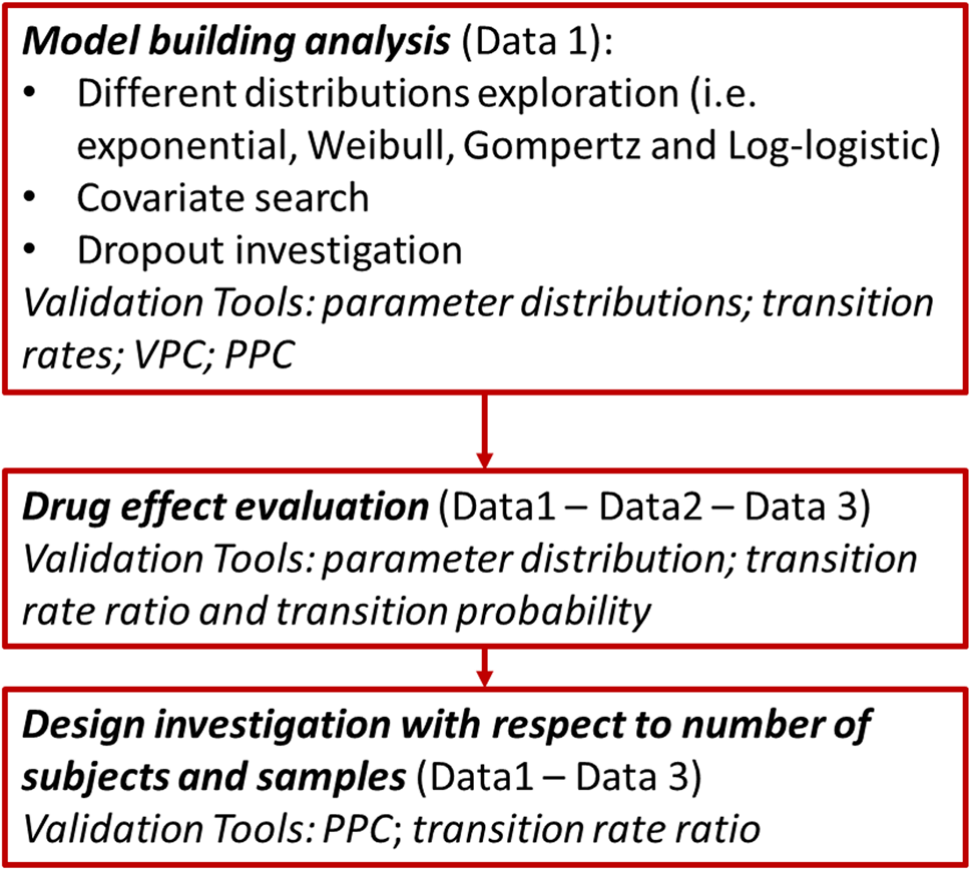
Schematic of the different analysis steps reporting which dataset and which tools/diagnostics were used

Figure 1 presents the schematic of all the analysis performed in this work reporting which dataset and which tools/diagnostics were used at each stage of the analysis. The results section is presented in the following way: the first part shows the different model building steps (i.e. test different distribution function, adding covariate, adding dropout model) that were performed using only the Data 1 dataset; the second part focuses on the drug effect identification in all the three datasets; the third part explores, using Data 3 and Data 1 respectively, the impact of different designs on the drug effect and a model evaluation on the ability to predict the outcome of longer duration trial (6–12 month) using only 3-month data.

### Model selection

All four models describing the semi-Markov transition process were tested, with both fixed and random effects on shape and scale parameters, the DIC and the shape parameters from these models are presented in Table 2. The log-logistic model according to DIC criteria seems to be the best among the four followed by the Weibull, the exponential model and finally the Gompertz model. Note that the random effect was tested in both shape (when present) and scale parameters and was found significant only on the scale parameter (DIC results not reported). A correlation term between alpha1 and alpha 2 in the log-logistic model was also tested by defining the random effects between the two states as realization of a multivariate normal distribution instead of two independent normal distributions but this attempt resulted in a non-significant drop of DIC. Trace plot from log-logistic model on the key parameters (alpha1, alpha2, theta1 and theta2) are presented in the supplementary material, Fig. S2.

**Table 2:** DIC, median and 95% credible intervals (CI) of the shape parameters (alpha1 and alpha2) of the four two-state mixed models

Model	DIC	Alpha_1_ - state 1 --> 2			Alpha_2_ - state 2 --> 1		
		Median	2.5	97.5	Median	2.5	97.5
Exponential	7236						
Weibull	7087	1.004	0.92	1.09	1.75	1.57	1.94
Gompertz	7247	− 9.12E − 04	− 2.59E − 03	7.76E − 04	− 5.99E − 03	− 1.19E − 02	− 4.97E − 04
Loglogistic	7026	1.34	1.22	1.48	2.97	2.67	3.27

*DIC* deviance information criterion

In Table 2 are also reported the shape parameters of the different distributions. Note that according to the log-logistic model, the transition rates over time are in both states having a bell shape increasing at the beginning and then decreasing towards zero when extrapolated to infinity, see Fig. S4. Note also that in state 2 the bell shape is less evident as only a relevant time window is presented (i.e. time that a subject can stay in symptomatic state). The simulation-based diagnostics relating to the log-logistic model are presented in Figs. S5–S7. Figure S5 shows that the log-logistic model yields a good performance as the observed values of the total observations, observations in state 1 and observations in state 2 are centred in their respective simulated distributions. In Fig. S6 the duration bins in both state 1 and state 2 seem to be captured by the log-logistic model reasonably well. In Fig. S7 the number of exacerbations is also well captured by the model.

#### Covariate selection

Table 3 presents the results of a covariate analysis performed using the log-logistic model as base model. Adding disease stage resulted in the greatest drop in DIC (4 points), even if the drop per se cannot be considered big. The parameter beta1 (β_1_ in Eq. 6), that represented the covariate effect of the disease stage in state 1, was significant for transitions from state 1 to state 2 [0 was not included in the 95% credible interval (CI)] and seemed to accelerate the transition from state 1 to state 2 (the scale parameter is decreasing due to the covariate contribution and as a consequence the sojourn time in state 1 is shorter). No other covariates had a similar drop of DIC, however, smoke status (also the more informative annual cigarette smoking packs) on parameter beta1 seemed to be responsible (0 was not included in the 95% CI) for slowing the transition from state 2 to state 1 (the scale parameter is increasing due to the covariate contribution and as a consequence the sojourn time in state 2 is longer). This suggests that if the patient is a current smoker the recovery from a COPD exacerbation would be slower and if a patient had a disease status that was moderate or severe, this would result in the patient transitioning to an exacerbation state faster. The baseline seasonality did not show any trends with respect to the covariate effect parameter. Also adding in an interaction between smoke and disease status didn’t improve the model fitting.

**Table 3:** DIC and covariate effect parameter (median and 95% CI) of the covariate analysis using the log-logistic model

Model	DIC	Beta1_1_ - state 1 --> 2			Beta1_2_ - state 2 --> 1		
		Median	2.5	97.5	Median	2.5	97.5
Loglogistic	7026						
Loglogistic + smoke	7025	0.07	− 0.30	0.42	0.26	0.08	0.44
Loglogistic + pky smoke	7025	− 0.002	− 0.01	0.01	0.005	0.001	0.01
Loglogistic + disease stage (ATS)	7022	− 0.40	− 0.78	− 0.05	− 0.11	− 0.29	0.09
Loglogistic + basaline seasonality	7028	− 0.08	− 0.44	0.29	− 0.06	− 0.25	0.12

*DIC* deviance information criterion

Figure S8 presents the simulation-based diagnostic for the log-logistic model with the inclusion of the disease stage covariate. The diagnostics suggest the model adequately described the observed data.

#### Drop out model

The DIC of the three investigated dropout models, applied only on dropout data, showed the log-logistic model was superior in describing the dropout data (i.e. DIC log-logistic = 1577; Weibull = 1579 and exponential = 3484). Disease stage was included as a covariate in the dropout model and was shown to be significant even though the drop in the DIC was around 3 points. In particular, the disease stage covariate effect was estimated with median value of − 0.68 (95% CI − 1.28, − 0.1). The simulation-based diagnostics relative to the dropout mechanism are depicted in Fig. S9 which shows the dropout rate was adequately captured based on the PPC and that the study length was also well captured based on the VPC plots; in particular, the simulations of subject study lengths that are dropping out are consistently reproduced.

### Final model (integrated two-state and drop out with covariate)

**Fig. 2:**
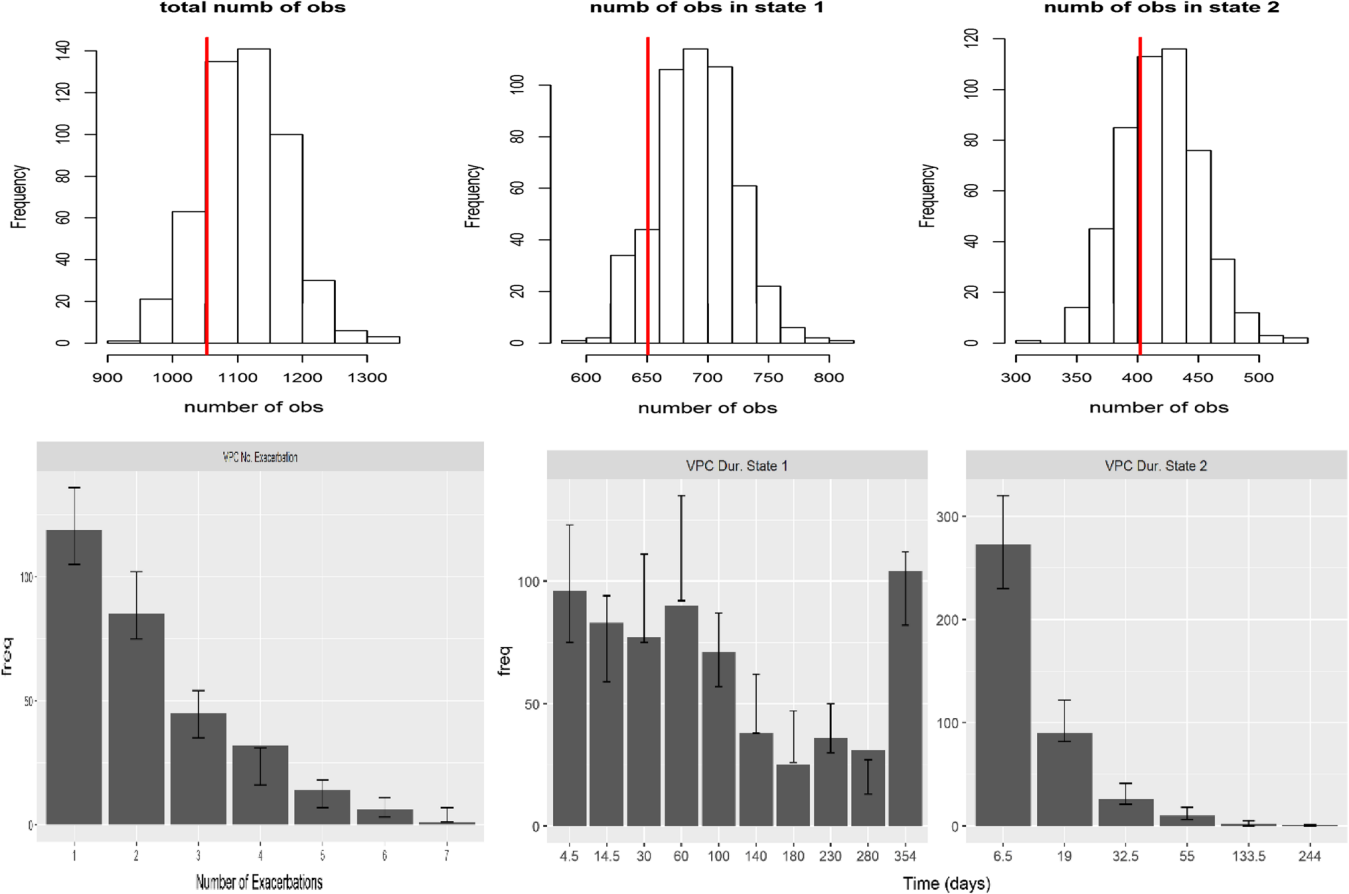
PPC on total number of observations, and number of observations in each state (top—red vertical line is the observed value) and VPCs on number of exacerbations (bottom left—solid bars are observed values, error bars are 95% CI obtained from model simulation), observations in state 1 (bottom middle) and observations in state 2 (bottom right) of the final integrated model (two-state model and the dropout mechanism with the inclusion of the disease stage covariate implemented with the log-logistic model) (Color figure online)

In Fig. 2 are presented the simulation-based diagnostics of the final model with the inclusion of the drop-out mechanism and the selected covariates. The performance of the final model was good as the observed pattern was reasonably reproduced by the simulated data. The introduction of the dropout model slightly increases the variability as shown by the wider confidence intervals (see for comparison Figs. S5–S7). Trace plots on the key parameters (alpha1, alpha2, theta1, theta2, beta1 and beta2) from the final model are presented in the supplementary material, Fig. S3.

### Drug effect evaluations

Table 4 presents the drug effects estimated on the three datasets involving drugs with different mechanisms of action using the final two-state log-logistic model. The drug effect is significant (i.e. the drug effect parameter posterior distribution does not include the 0 value) on different transitions (i.e. from asymptomatic to symptomatic or vice versa). For Data 1 (placebo vs Salmeterol), Data 2 (VI vs all doses of FF/VI) and the low EOS sub-group of data3(placebo vs highest dose of Losmapimod) the drug effect was statistically significant in the transition from asymptomatic to symptomatic state and the drug acts to slow down the transition rate towards the exacerbation state. For Data 3 [placebo and 2 smaller doses vs highest dose (i.e. 15 mg)] the drug effect is significant on the opposite direction (i.e. from symptomatic to asymptomatic state) and it acts to speed up the transition towards the non-exacerbated state. Note that the model used for fitting Data 3 was simplified; the random effect relative to state 2 was deleted because the data were not able to support it as the study length is 6 months with lower exacerbation rate compared to Data 1 and Data 2. Note also that for this dataset the dropout window that defines if the patient truly dropout was reduced to 2 weeks.

**Table 4:** Estimated drug effects (median and 95% CI) using the log-logistic two-state mixed model

Dataset	Beta1_1_ - state 1 --> 2			Beta1_2_ - state 2 --> 1		
	Median	2.5	97.5	Median	2.5	97.5
Data 1	0.310	0.046	0.559	− 0.031	− 0.162	0.104
Data 2	0.224	0.0933	0.353	0.025	− 0.037	0.087
Data 3	0.211	− 0.229	0.651	− 0.196	− 0.396	− 0.002
Data 3 low EOS	0.818	0.020	1.740	− 0.110	− 0.430	0.223

**Fig. 3:**
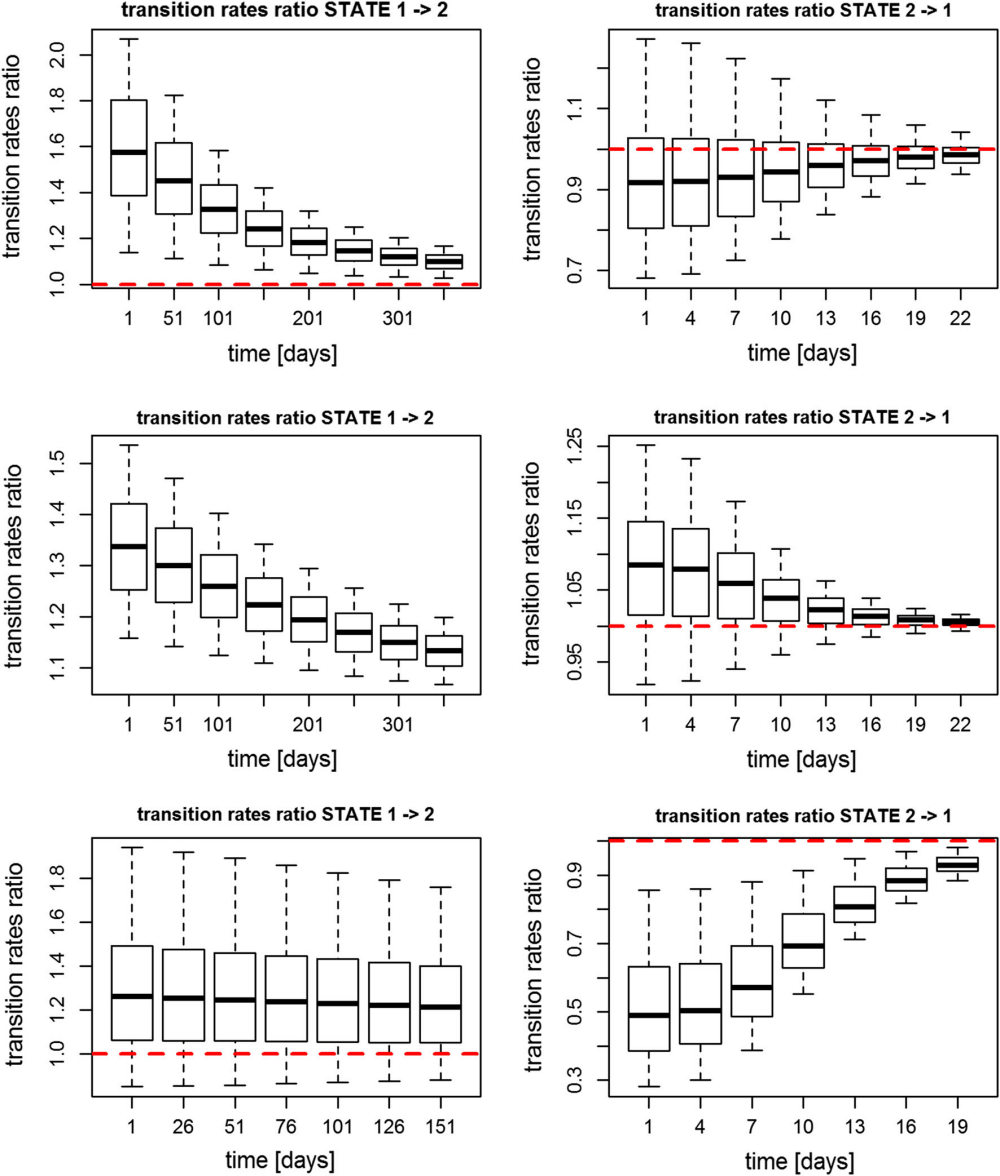
Transition rate ratios (placebo/active) using the log-logistic model in Data 1 (on the top), Data 2 (in the middle) and Data 3 (on the bottom)

**Fig. 4:**
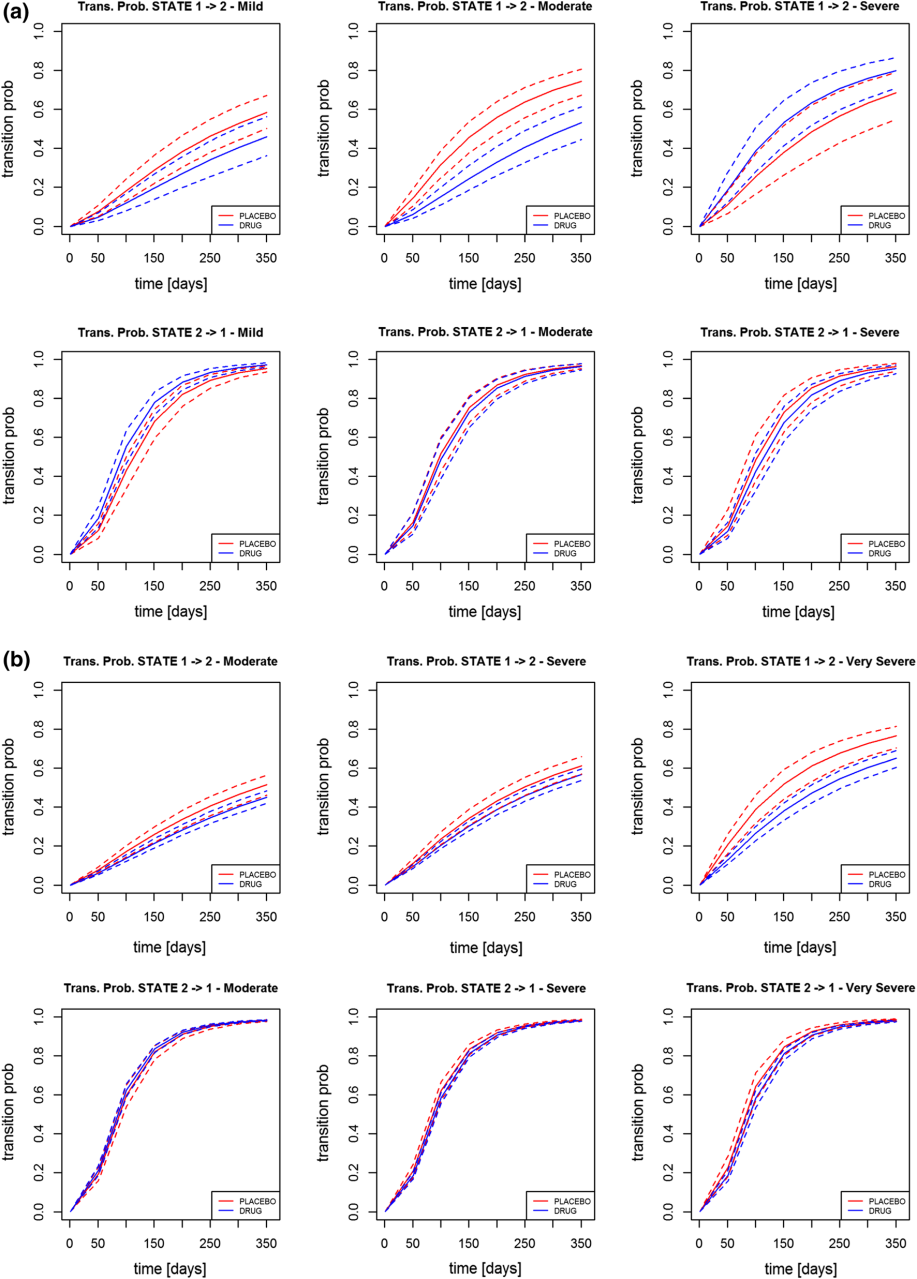

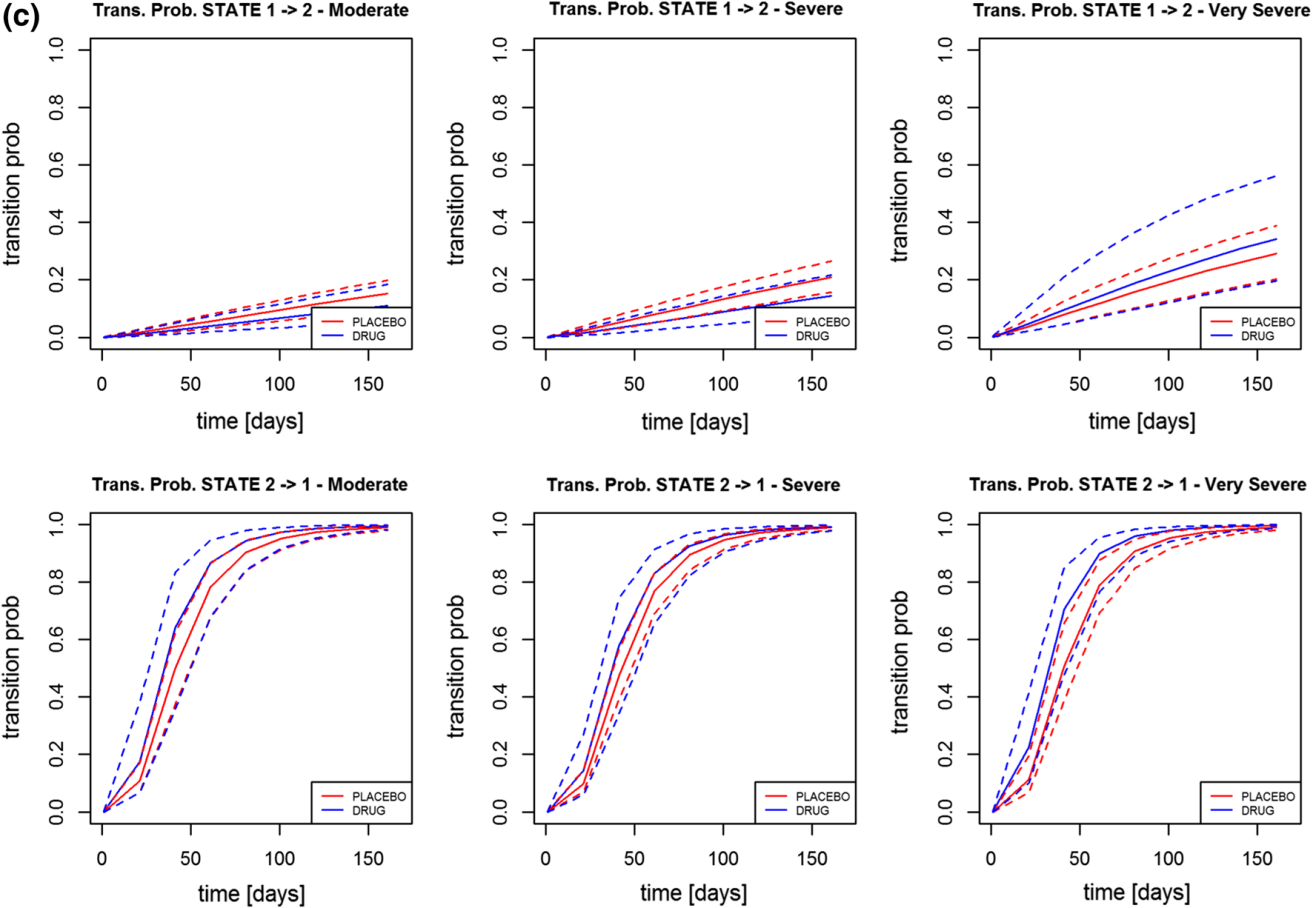
Transition probability using the log-logistic model for different disease stage level in Data 1 (**a**), in Data 2 (**b**) and in Data 3 (**c**) [red lines—placebo arm; blue lines—treatment arm] (Color figure online)

In Figs. 3, 4 are presented for the log-logistic model the transition rates ratios (placebo/active) over time and the transition probability stratified by disease severity for all the three datasets. As far as Data 1 is concerned, the transition rate ratio is significantly different from one in the transition from asymptomatic to symptomatic state and by being greater than one it suggests that the drug is acting in slowing down the passage from asymptomatic to symptomatic state. This ratio is decreasing over time suggesting that the longer the patient stays in the non-exacerbated state the more unlikely is that he will have exacerbations in both placebo and drug arms. The transition probability stratified by severity shows that the drug is acting mainly in the moderate severity as the transition rates are clearly separate. Similar observations were shown for Data 2 and the relative transition rates ratio over time and the transition probability stratified by severity. This time, the severity stratification is significant for the most severe patients. As far as Data 3 is concerned, there is a significant difference in transition from exacerbated state (state 2) to non-exacerbated state (state 1), as the ratio was smaller than one it suggested that the drug was acting in speeding up the passage from symptomatic to asymptomatic state. The transition probability instead is not significant for any of the severity levels.

### Design considerations

**Fig. 5:**
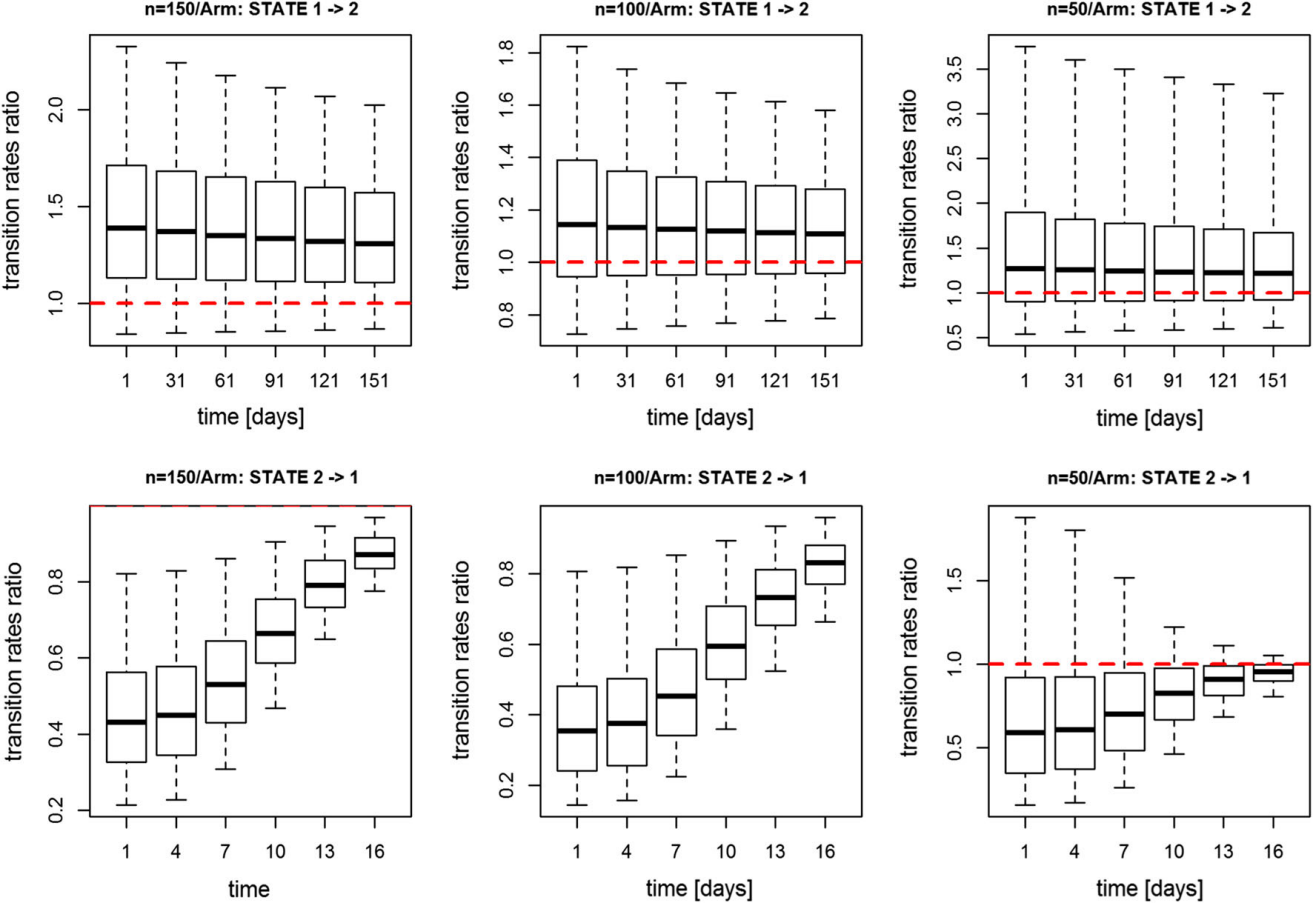
Transition rates ratio using log-logistic model for Data 3 using respectively 150, 100 and 50 subjects per arm (from left to right)

**Fig. 6:**
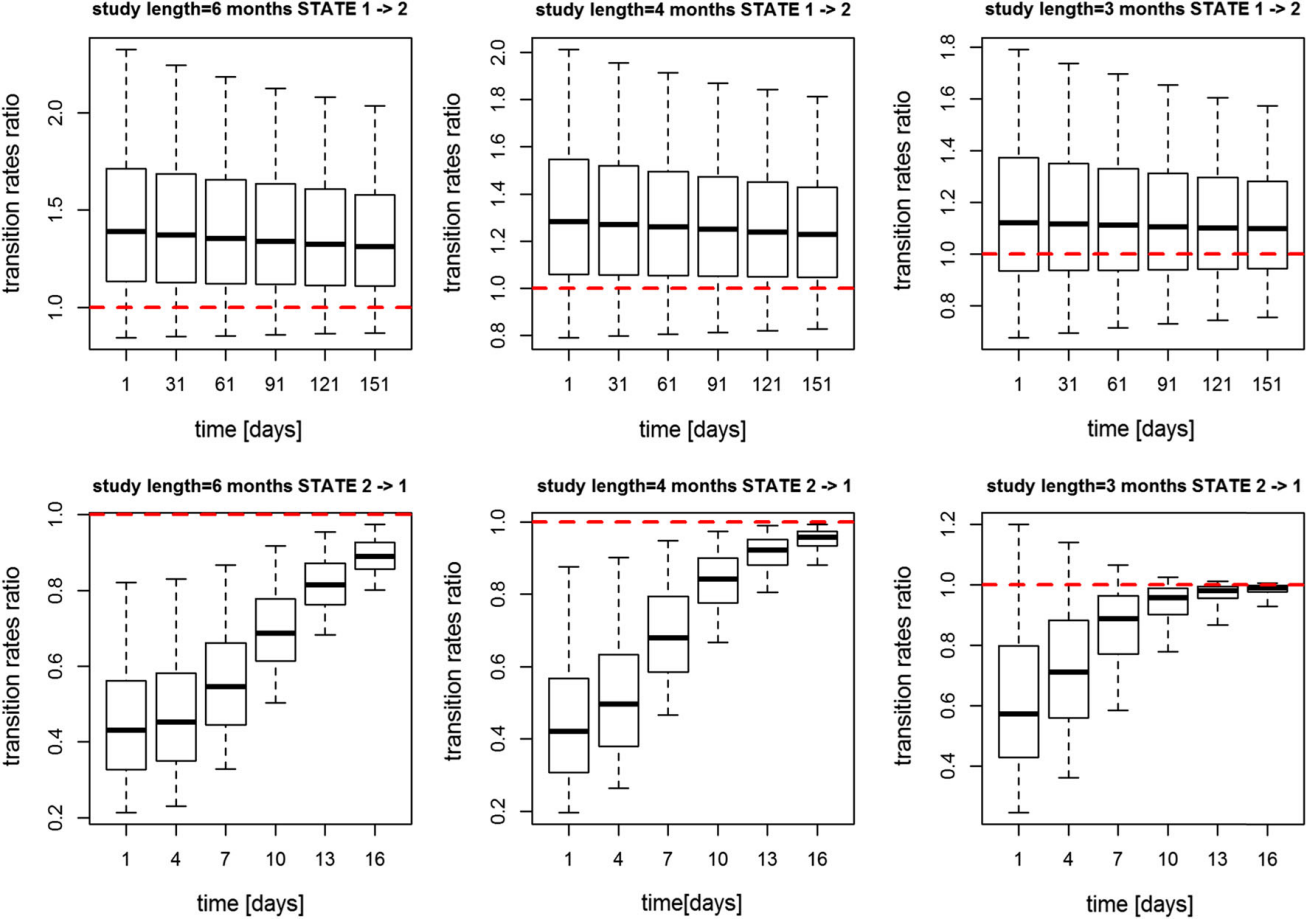
Transition rates ratio using log-logistic model for Data 3 using study length equal to 168, 120 and 90 days (from left to right), respectively and keeping 150 subjects per arm

In Figs. 5, 6 are presented transition rates ratios over time (placebo vs highest dose) using Data 3 under different design settings. In particular, in Fig. 5, the sample size was reduced from 150 to 100 and to 50, but assuming the study length was 6 months. The transition rates ratio was significantly different from 1 up to a sample size of 100 subjects per treatment group. In Fig. 6, the sample size was kept as equal to 150 whereas the study length was cut from approximately 6 to 4 and to 3 months (i.e. 168, 120 and 90 days): in this case the transition rates ratio was no longer significantly different from 1 with study duration of 3 months.

**Fig. 7:**
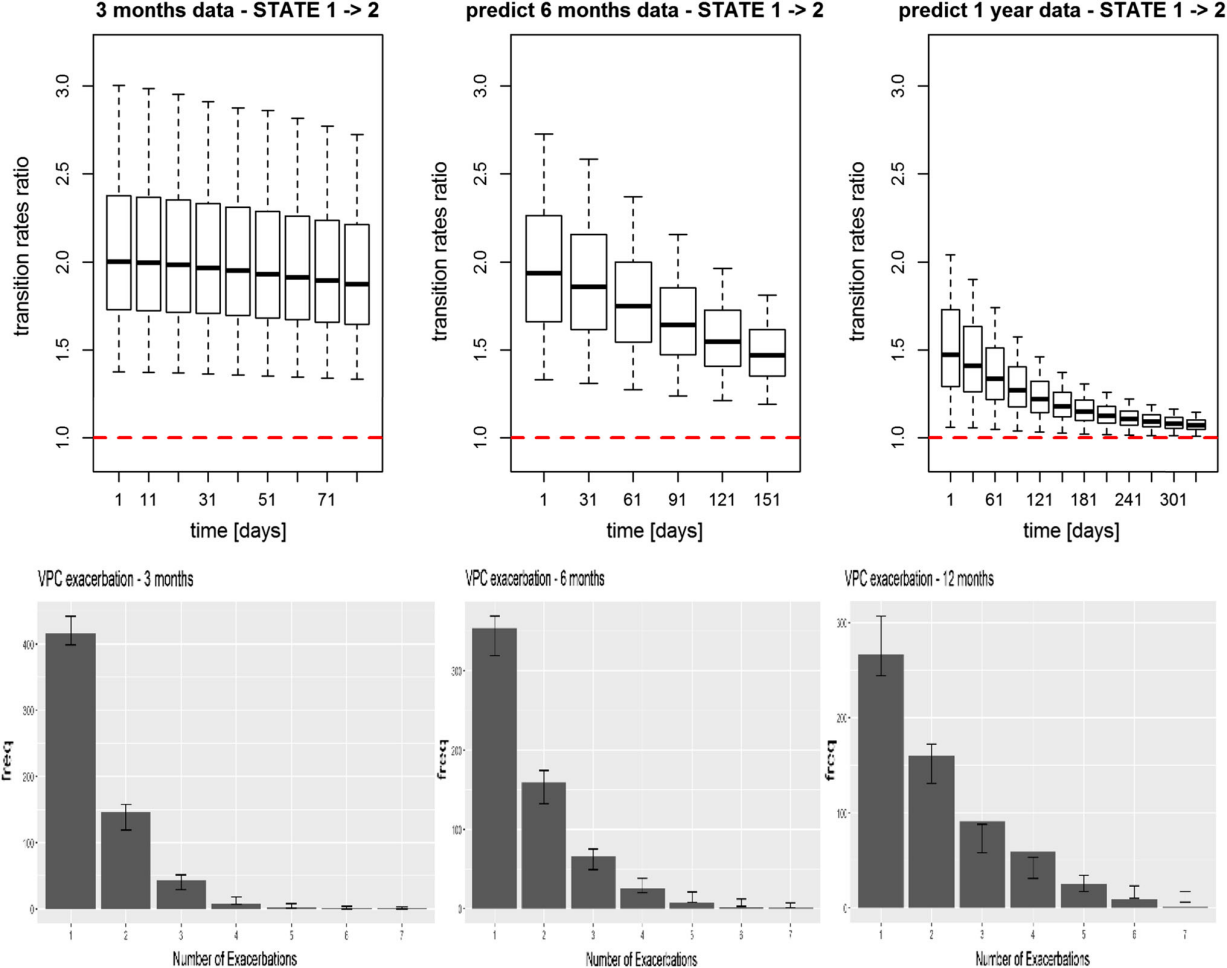
Transition rates ratio (on top) and number of exacerbations (on the bottom—solid bars are observed values, error bars are 95% CI obtained from model simulation) using log-logistic model for Data 1 extrapolating to 6 months (middle) and 1 year (right) from estimates of 3-months data (left)

The extrapolation capability of the model was tested using Data 1 (Fig. 7). In particular, using data from 3 months’ trial, the model predictions at 6 and 12 months were compared to the observed data. The longer the extrapolation period from 3 months to 1 year, the more the model predictability is poor as the VPC plots of the number of exacerbations show in Fig. 7—bottom panel (i.e. the CI become wider and less centred on the observed value) and the less the drug effect is well characterized as shown in the upper panel of Fig. 7 (i.e. the transition rates ratio gets closer to identity line).

## Discussions

Exacerbations are historically analysed in terms of frequency through the negative binomial approach but due to the high variability in occurrence and duration of the event this frequency based approach seems to be not optimal. A more complete use of the data information is to consider the time that the patient spends in the exacerbated (symptomatic) and non-exacerbated (asymptomatic) state. Four different models were then tested to describe the transition process between the asymptomatic and symptomatic state as a first order Markov (exponential model) or a first order semi-Markov [Weibull (as Cook et al.), Gompertz and log-logistic models]. These models differ in the assumptions on their hazard or survival functions (or a transformation of these functions) with time.

Using the DIC criteria, log-logistic model was selected as the best model to describe the data and with its semi-Markov approach resulted in better description of the process compared to the Markov approach proposed in Cook’s et al. (i.e. lower DIC than the Weibull model) suggesting that that the memory of the exacerbation process is not constant in time. It is notable that for this work, the transition models between state 1 and 2 were assumed to be the same, but allowing different parameters to be estimated. In theory, it is possible to select different models for state 1 and 2. A preliminary testing showed that it might be possible a model with Weibull distribution for transitions from state 1 to 2, and with log-logistic for transitions from state 2 to 1 could be equally valid. Such evaluations may be considered in future. A final consideration to make is that the underlying exacerbation process is not known and that the log-logistic distribution was chosen only accordingly to model fit criteria rather than biological based information so further data need to be collected in the future to confirm this hypothesis. Note that potentially all data could have been analysed simultaneously instead of utilising separately Data 1 for the investigation of the base model and in a second stage the study information could have been added as a covariate to better capture the data.

Different non-time varying covariates were tested and the most significant was the disease stage. Possible future exploration might include time varying covariates such as season during the trial and exacerbation severity.

The dropout mechanism was assumed to follow a missing-at-random (MAR) pattern and was found to be better described by a log-logistic distribution. This choice was reasonable because the observed dropout rate due to exacerbations was low [e.g. for Data 2 less than 3% (97 subjects) and for Data 3 less than the 2% (12 subjects) (13, 14)] and because the majority of the dropout was caused by adverse events that justify further the choice of independency of the mechanism from efficacy.

It is worthy to note that the covariate inclusion and the dropout model in the final model did not dramatically improve the model performance as can be seen in the simulation-based diagnostics (see Figs. S5–S7, S8 and Fig. 2). This can be explained by the fact that the covariate that was included in the model did not create a big drop in the DIC and by the fact that the dropout values observed in the dataset were really small, almost negligible.

The analysis of the three datasets revealed that the two-state model was able to identify the drug effect in heterogeneous COPD populations (i.e. different disease severity and exacerbations rates) involving drugs with different mechanism of action. It is noteworthy that for Data 1, Data 2 and the low EOS subset of Data 3, the two-state model confirmed the results reported using the standard negative binomial models (13, 14) and, in addition, for Data 3 the two-state model was able to detect a drug effect that was not significant using the negative binomial. An additional advantage of the two-state model was to identify potential drug effect showing increased transition rate from state 2 to state 1. Note that with the standard negative binomial approach the direction of the drug action would not be identifiable. Moreover, the analysis performed on the drug interaction with the severity of the patient showed that for data 1 the drug effect was relatively stronger for moderate to severe patients (transition probabilities of the placebo arm were separated and higher than the treatment arm), whereas in the Data 2, drug effect was larger in the sub-group of very severe patients, see Fig. 4. This result is of particular interest as it may help to identify the population in which the drug may be more efficacious but as no mechanistic explanation is available further investigation should be done to validate the finding.

A useful application of the final model was to evaluate drug effect under different study designs as shown in Figs. 5, 6. Using Data 3 we observe that reducing the study duration up to 4 months or reducing the number of subjects up to 100 per arm would not compromise the detection of a drug effect. This is an important result as it suggests it would be possible to shorten the study length and so reduce the trial cost without compromising any drug effect identification.

The extrapolation capability of the two-state log-logistic model to 6 months and 12 months using parameter estimates obtained from Data 1 with only 3 months data was reasonable, see Fig. 7. The predictions at 12 months were slightly deteriorated but overall the estimated drug effect at 1-year was well predicted using only 3 months’ data. Therefore, these results suggest that the model can reliably be used in simulation framework to explore the compound behaviour in different drug development setting.

Note that these design investigation findings are difficult to generalize as they are bounded to the dataset under analysis (i.e. dataset rate of exacerbation; size of the drug effect), so further efforts need to be undertaken in the future for model refinement.

## Conclusions

In this work, we expanded the Cook’s two-state model by investigating different semi-Markov transition models using three clinical studies with COPD exacerbations data. The log-logistic model adequately characterized the duration and number of COPD exacerbations, as well as capturing the effect of different treatment interventions (i.e. the drug effect was detected in both directions—slowing down transition to exacerbation state and speeding up transition to non-exacerbated state). Preliminary design investigations with actual study data showed that, given the dataset under analysis (e.g. specific rate of exacerbation and identified drug effect), a clear drug effect can be detected even with shorter study duration (i.e. from 6 to 4 months) or relatively lower sample size (i.e. from 150 subjects to 100).

## Electronic supplementary material

Below is the link to the electronic supplementary material.

Supplementary material 1 (PDF 5376 kb)

## Data Availability

Anonymized individual participant data from these studies plus the annotated case report form, protocol, reporting and analysis plan, data set specifications, raw dataset, analysis-ready dataset, and clinical study report are available for research proposals approved by an independent review committee. Proposals should be submitted to www.clinicalstudydatarequest.com. A data access agreement will be required.
