# Correction to: Bayesian approach to investigate a two-state mixed model of COPD exacerbations

**DOI:** 10.1007/s10928-019-09664-1

**Published:** 2019-11-15

**Authors:** Anna Largajolli, Misba Beerahee, Shuying Yang

**Affiliations:** 1 GlaxoSmithKline, Research and Development, Uxbridge, UK; 2 Present Address: Certara Strategic Consulting, Via G.B. Pirelli 27, 20124 Milano, Italy; 3 Clinical Pharmacology Modelling and Simulation, Quantitative Sciences, GlaxoSmithKline, Stockley Park West, 1-3 Ironbridge Road, Uxbridge, Middlesex UB11 1BT UK

## Correction to: Journal of Pharmacokinetics and Pharmacodynamics (2019) 46:371–384 10.1007/s10928-019-09643-6

The article “Bayesian approach to investigate a two-state mixed model of COPD exacerbations”, written by Anna Largajolli, Misba Beerahee, Shuying Yang, was originally published electronically on the publisher’s internet portal (currently SpringerLink) on 13 June 2019 without open access.

With the author(s)’ decision to opt for Open Choice the copyright of the article changed on November 2019 to © The Author(s) 2019 and the article is forthwith distributed under the terms of the Creative Commons Attribution 4.0 International License (http://creativecommons.org/licenses/by/4.0/), which permits use, duplication, adaptation, distribution and reproduction in any medium or format, as long as you give appropriate credit to the original author(s) and the source, provide a link to the Creative Commons license and indicate if changes were made.

