# Supplementary material for: Bayesian approach to investigate a two-state mixed model of COPD exacerbations
Source: J Pharmacokinet Pharmacodyn. 2019 Jun 13;46(4):371–84. doi: 10.1007/s10928-019-09643-6 (PMC6848253; doi:10.1007/s10928-019-09643-6)

## *SUPPLEMENTARY MATERIAL*

## Supplementary Figures

Figure S1. Plot of some representative subjects of the sojourn in state 1 (red colour) and sojourn in state 2 (blue colour)

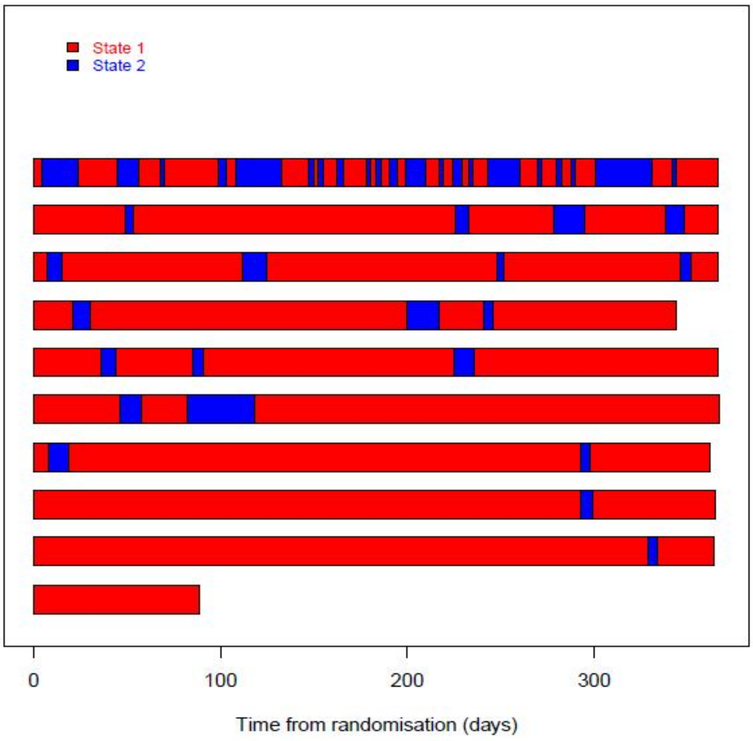


Figure S2: Trace plots of Key Parameters: shape (logalpha[1]=log(alpha1), logalpha[2]=log(alpha2)) and scale parameter (logtheta0[1]=log(theta1), logtheta0[2]=log(theta2)) in the base log-logistic model

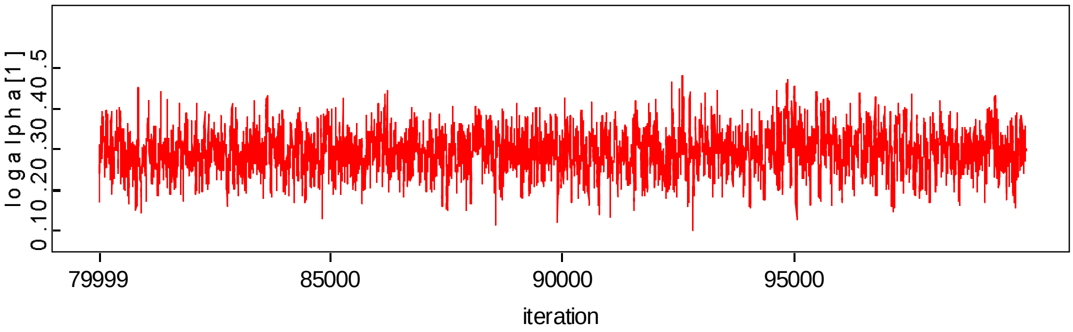

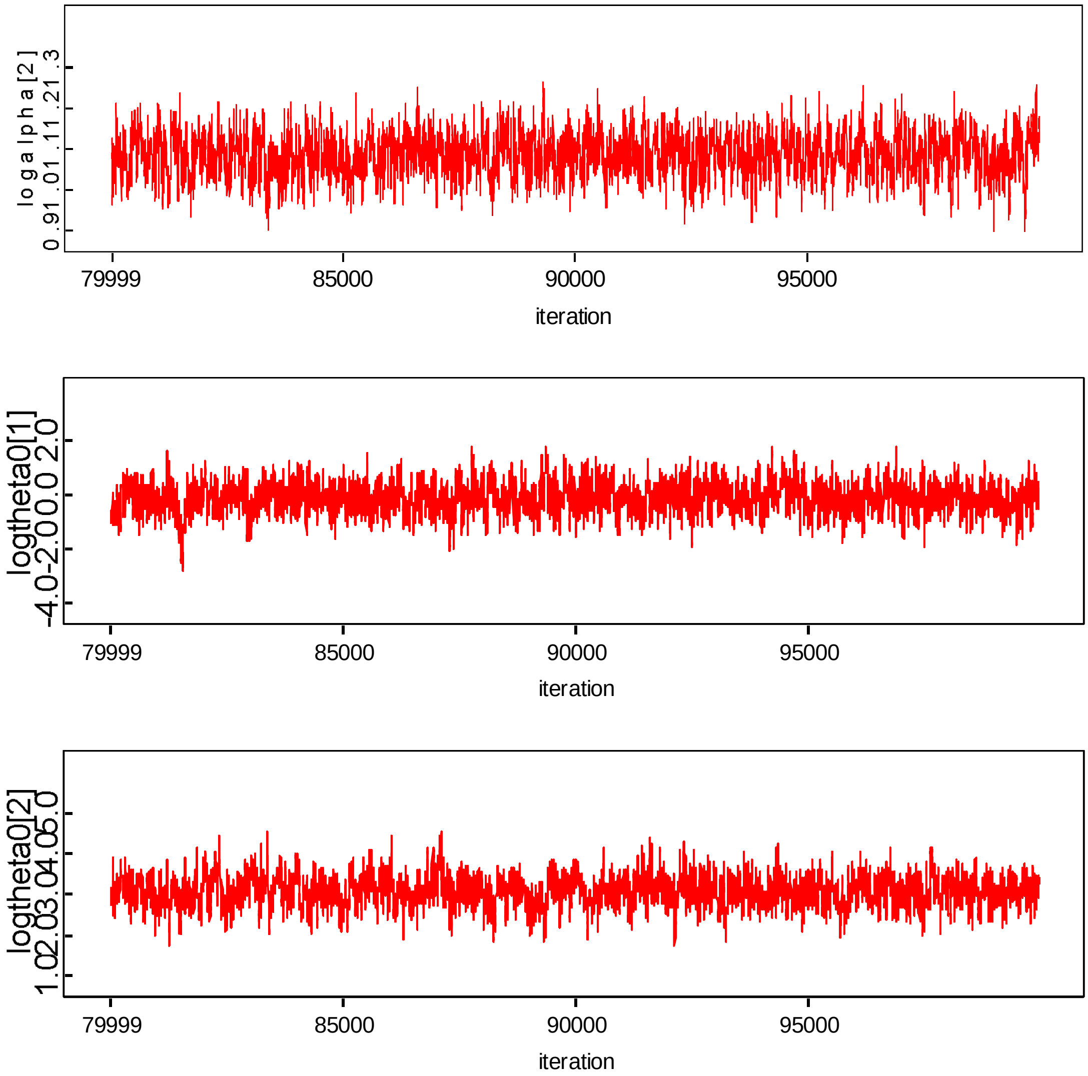


Figure S3: Trace plots of Key Parameters in the final log-logistic model: shape (alpha[1]=alpha1, alpha[2]=alpha2 and alpha[3]=alpha$_{dropout}$), scale parameter (logtheta0[1]=log(theta$_0$1), logtheta0[2]=log(theta$_0$2) and logtheta0[3]=log(theta$_{0-dropout}$)) and covariate effect (beta1[1]=beta$_1$1, beta1[2]=beta$_1$2 and beta1[3]=beta$_1$ $_{dropout}$)

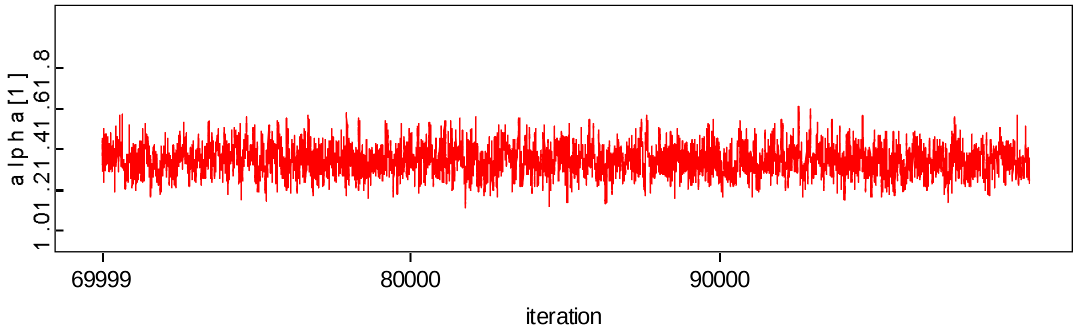

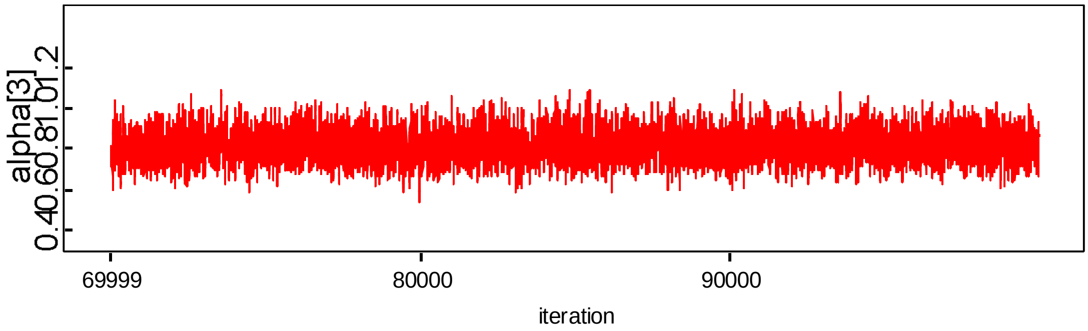
alpha[3]
0.4 0.6 0.8 1.0 1.2
69999
80000
90000
iteration

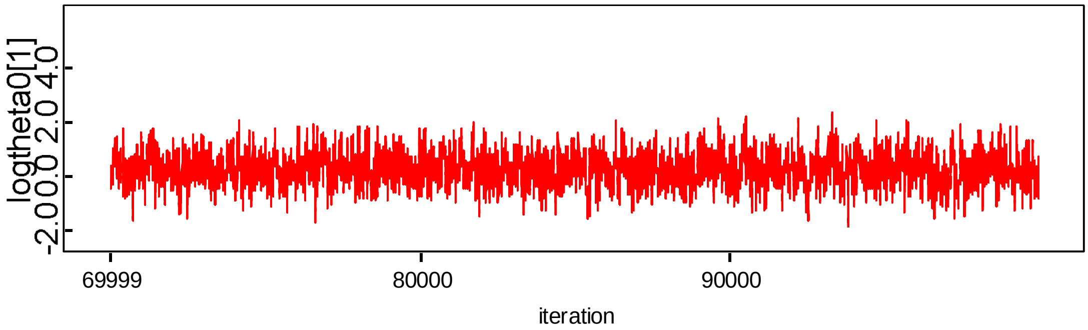
logtheta0[1]
-2.0 0.0 2.0 4.0
69999
80000
90000
iteration

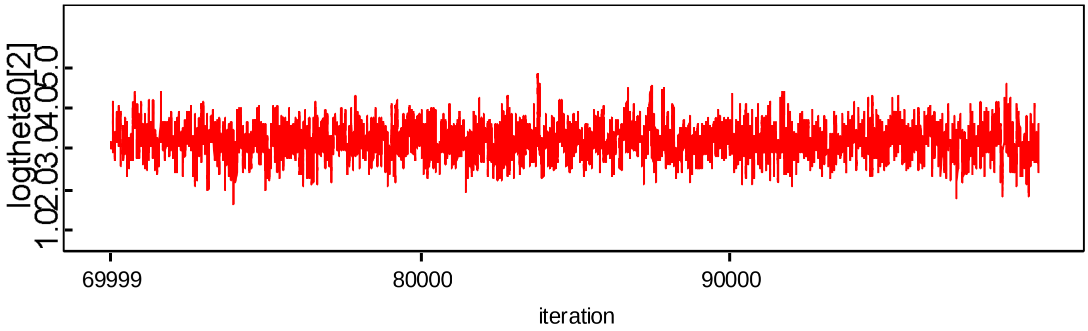
logtheta0[2]
1.0 2.0 3.0 4.0 5.0
69999
80000
90000
iteration

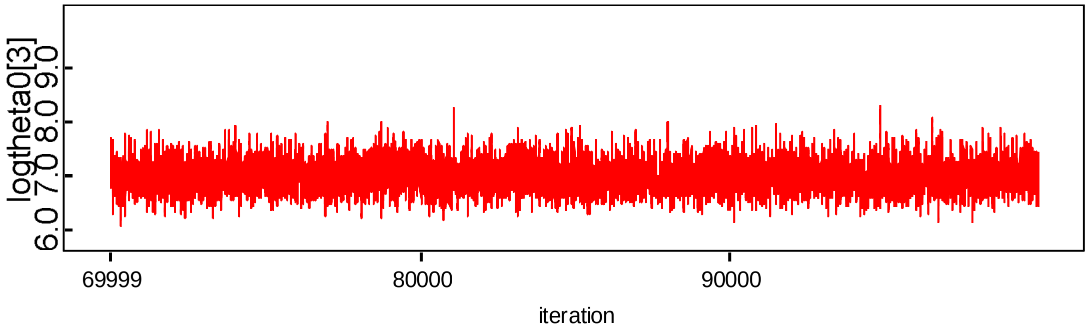
logtheta0[3]
6.0 7.0 8.0 9.0
69999
80000
90000
iteration

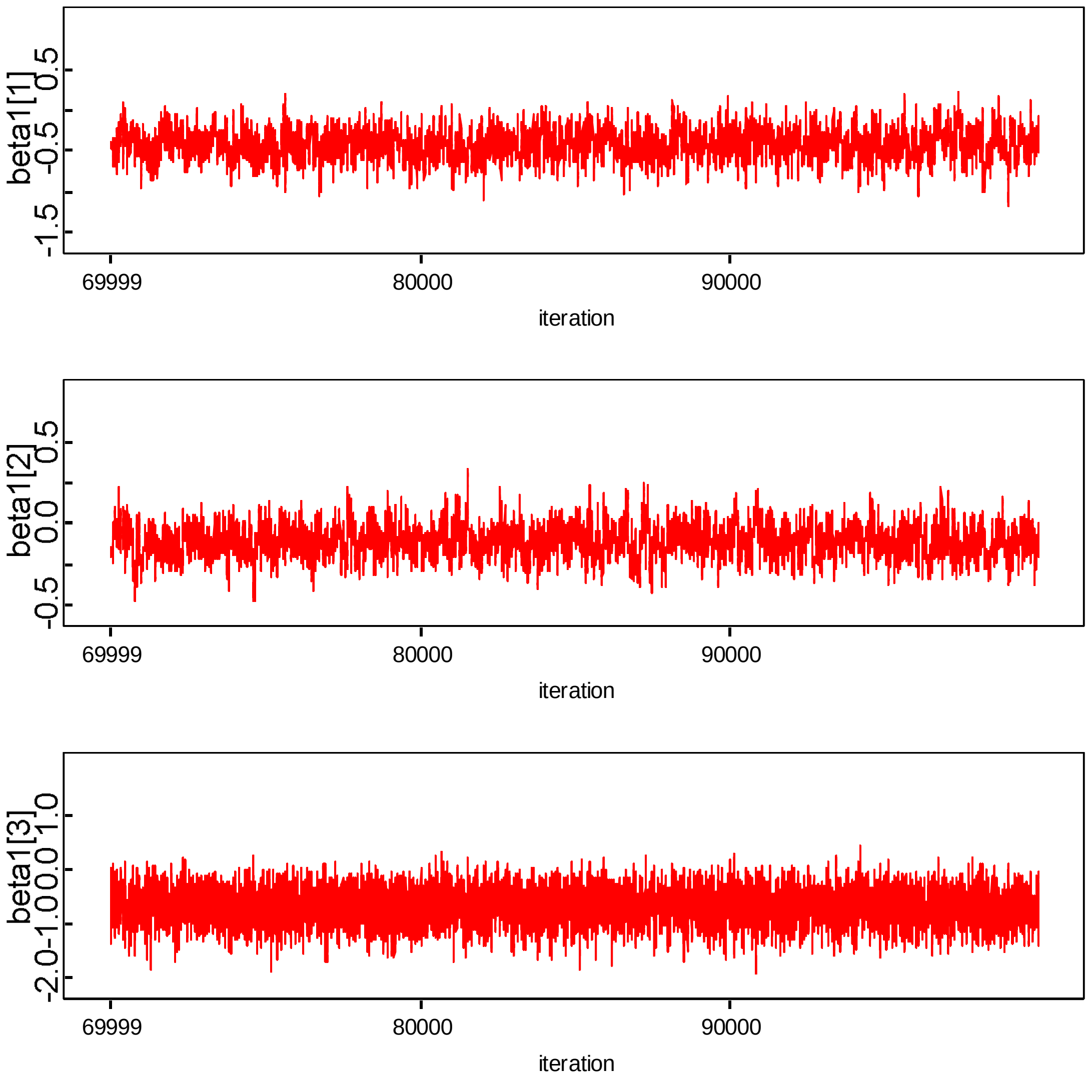


Figure S4. Transition rates over time of the log-logistic model from state 1 (on the left) and from state 2 (on the right)

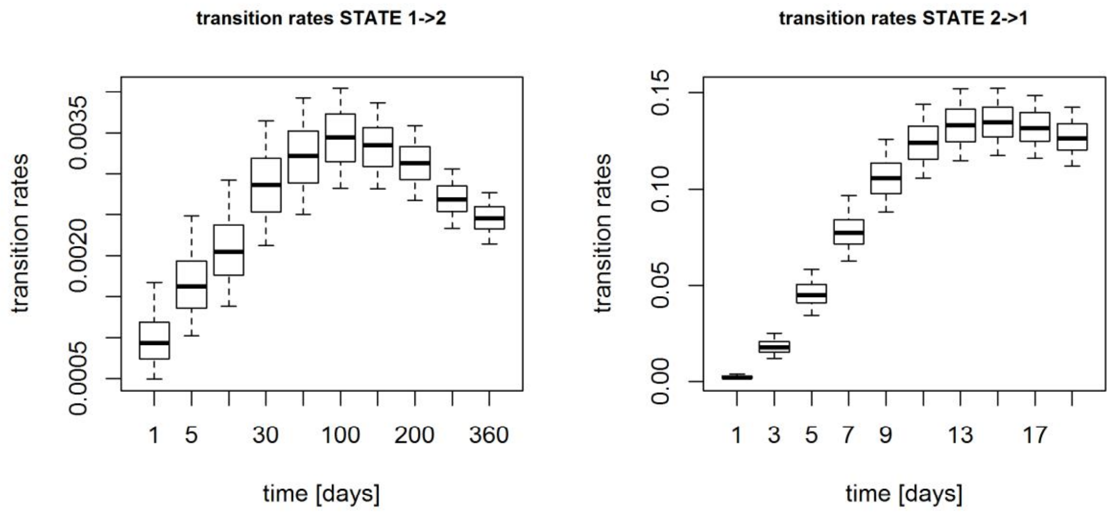

Figure S5. PPC of log-logistic model (left panel - with respect to total number of observations; middle panel - with respect to number of observations in state 1; right panel - with respect to number of observations in state 2); red vertical line is the observed value

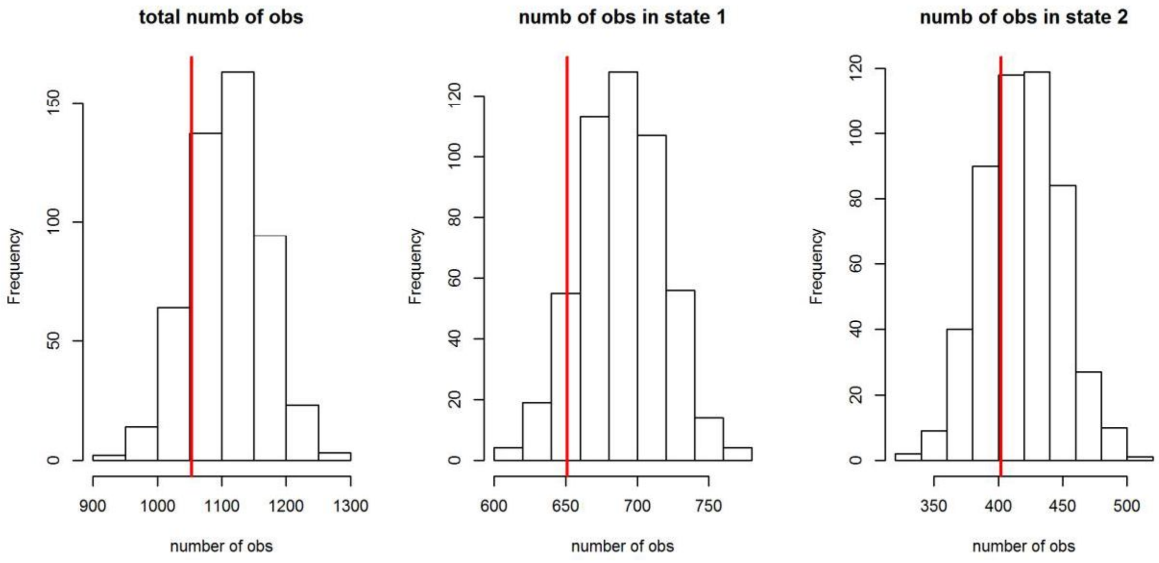


Figure S6. VPC of duration from state 1 (on the left) and state 2 (on the right) with log-logistic model (solid bars are observed values; error bars are 95% CI obtained from model simulations)

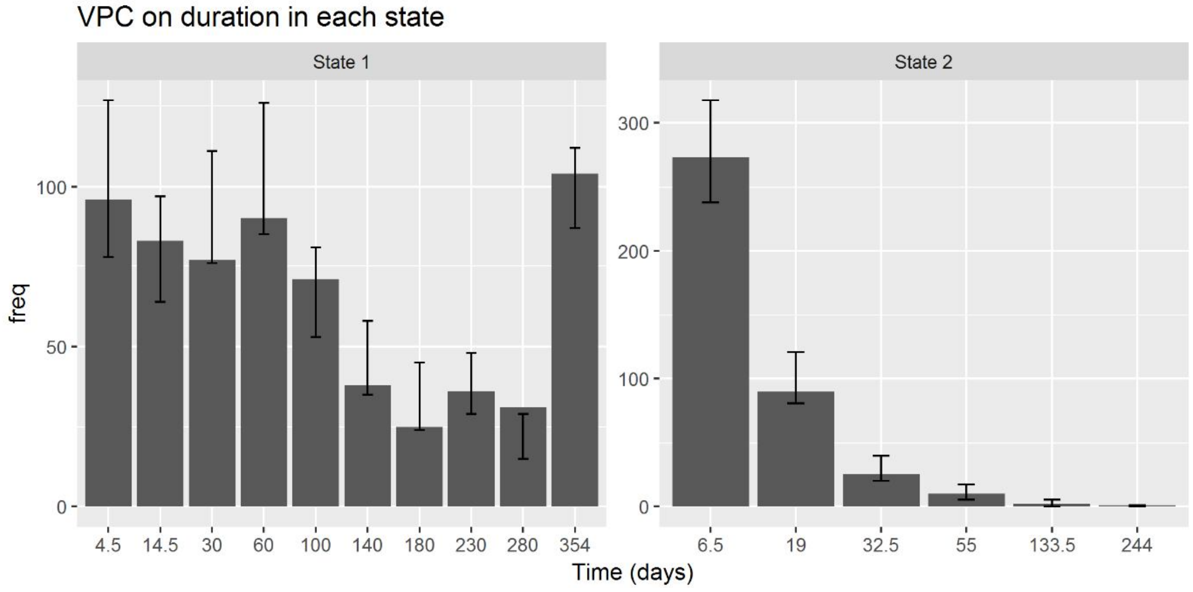

Figure S7. VPC of log-logistic model on number exacerbations (solid bars are observed values; error bars are 95% CI obtained from model simulations)

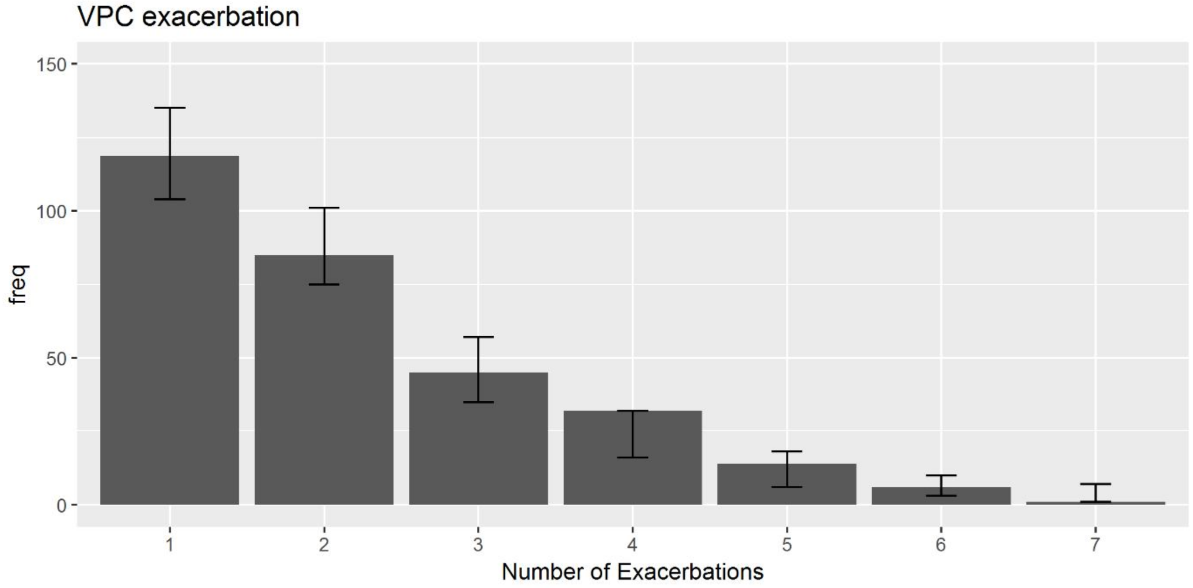


Figure S8. PPC (top left - red vertical line is the observed value) and VPCs on number of exacerbations (bottom - solid bars are observed values, error bars are 95% CI obtained from model simulation), observations in state 1 (middle left-solid bars are observed values, error bars are 95% CI obtained from model simulation) and observations in state 2 (middle right) of the log-logistic model with the inclusion of the disease stage as a covariate

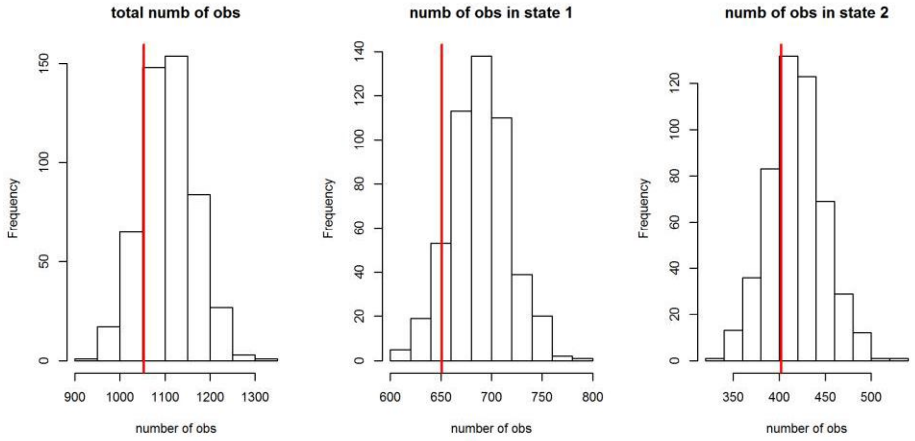

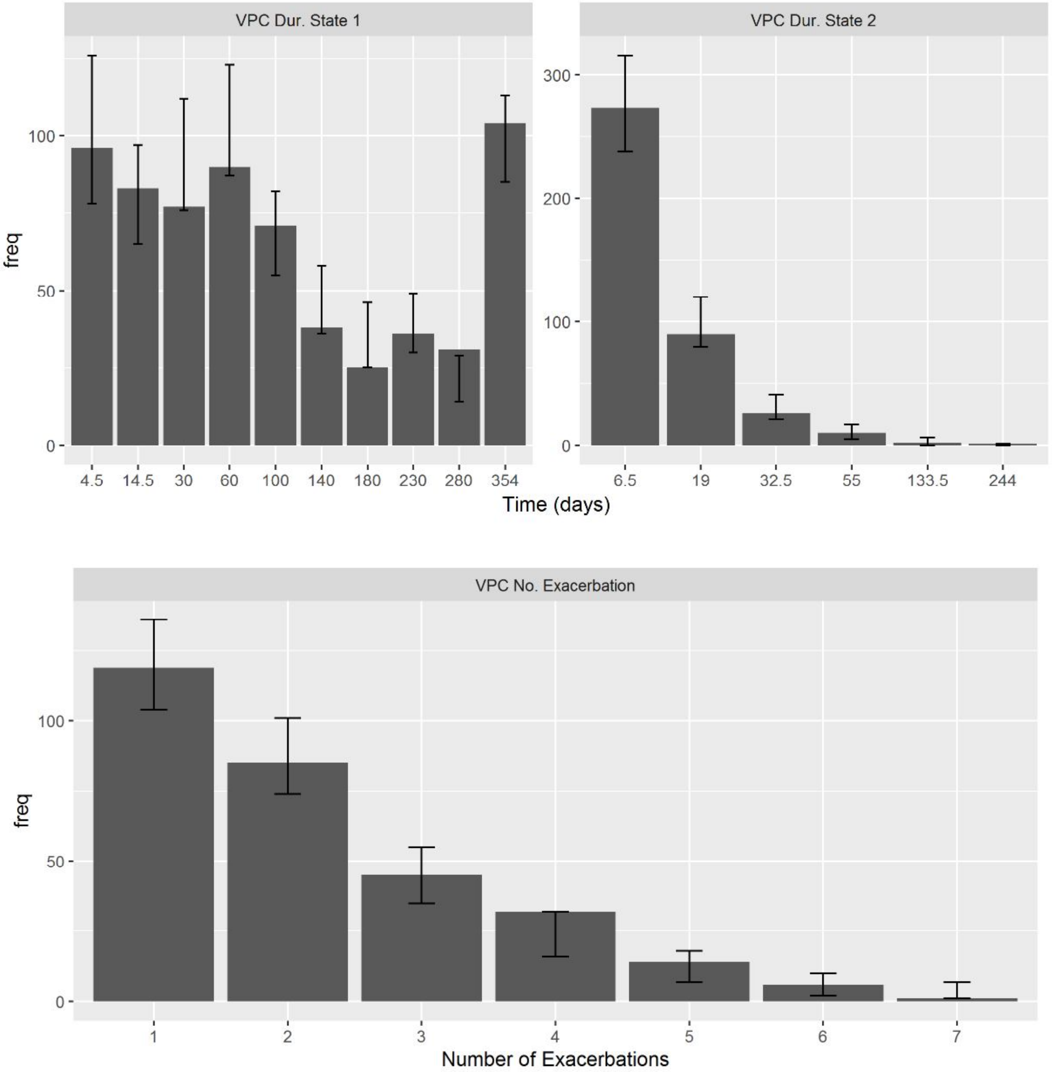
VPC Dur. State 1
VPC Dur. State 2
freq
Time (days)
4.5
14.5
30
60
100
140
180
230
280
354
6.5
19
32.5
55
133.5
244
VPC No. Exacerbation
Number of Exacerbations

Figure S9. PPC of dropout rate (on the left - red vertical line is the observed value) and VPC (on the right- (solid bars are observed values, error bars are 95% CI obtained from model simulation)) of study length of subjects that are dropped out using the Log-logistic model with the inclusion of disease stage covariate

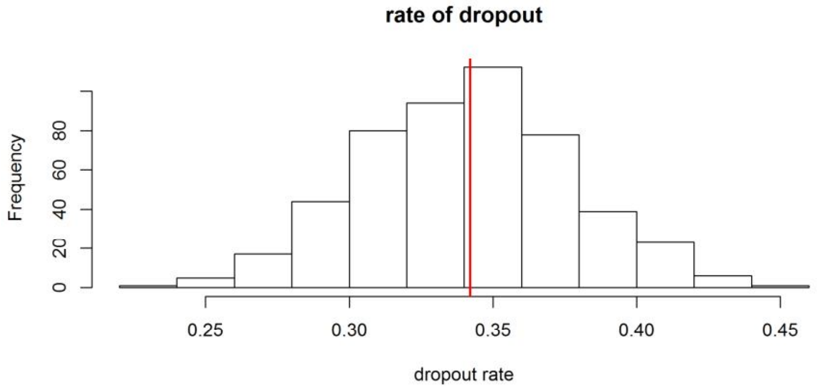


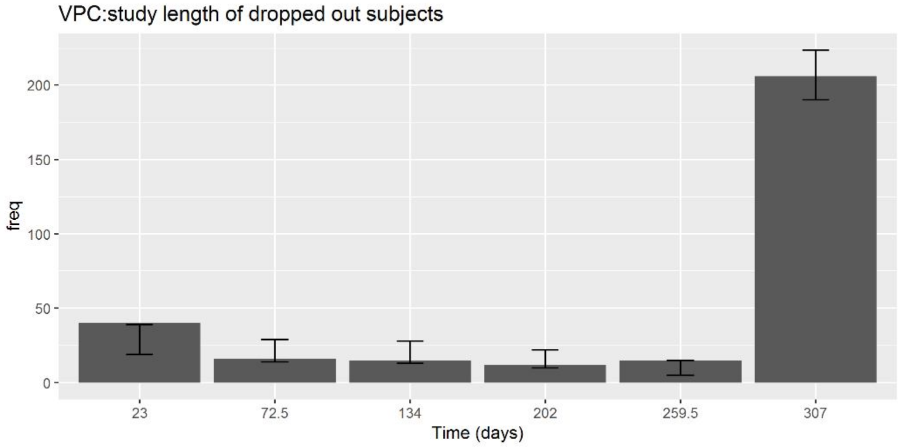

Supplement: Supplementary file 1 — Supplementary material 1 (PDF 5376 kb) [file 10928_2019_9643_MOESM1_ESM.pdf]
